# Association of metabolic syndrome and its components with the risk of urologic cancers: a prospective cohort study

**DOI:** 10.1186/s12894-023-01324-4

**Published:** 2023-09-22

**Authors:** Runxue Jiang, Xia Wang, Zhi Li, Haifeng Cai, Zhiguo Sun, Shouling Wu, Shuohua Chen, Hailong Hu

**Affiliations:** 1 Department of Oncology Surgery, Tangshan People’s Hospital, No.65 Shengli Road, Tangshan, 063000 China; 2 Department of Urology, Tianjin Institute of Urology, The Second Hospital of Tianjin Medical University, No.23 Pingjiang Road, Tianjin, 300211 China; 3 Department of Gynaecology, Tangshan Hongci Hospital, Tangshan, 063000 China; 4 Health Department of Kailuan (Group), Tangshan, 063000 China

**Keywords:** Urologic cancer, Metabolic syndrome, Cohort study

## Abstract

**Objective:**

To investigate the association between metabolic syndrome (MetS) and its components and the risk of developing urologic cancers.

**Methods:**

This study included 101,510 observation subjects from May 2006 to December 2007. The subjects received questionnaires and were subjected to clinical and laboratory examinations to collect data on baseline population characteristics, waist circumference (WC), blood pressure (BP), blood glucose, blood lipids, lifestyle, and past disease history. Finally, follow-up was conducted from the date of recruitment to December 31, 2019. Cox proportional hazards modelling was applied to analyze the association between MetS and its components and the risk of developing urologic cancers.

**Results:**

A total of 97,975 observation subjects met the inclusion criteria. The cumulative follow-up period included 1,209,178.65 person-years, and the median follow-up time was 13.03 years. During the follow-up period, 485 cases of urologic cancers (165 cases of kidney cancer, 134 cases of prostate cancer, 158 cases of bladder cancer, and 28 cases of other urologic cancers) were diagnosed. The log-rank test results for the cumulative incidences of urologic cancer, kidney cancer, and prostate cancer indicated significant (*P* < 0.01) differences between the MetS and non-MetS groups (0.70% vs. 0.48%, 0.27% vs. 0.15%, and 0.22% vs. 0.13%, respectively). Compared to the non-MetS group, the risk of developing urologic [HR (95% CI) = 1.29 (1.08–1.55)], kidney [HR (95% CI) = 1.74 (1.28–2.37)], and prostate [HR (95% CI) = 1.47 (1.04–2.07)] cancers was significantly higher in the MetS group. In the MetS group, elevated BP increased the risk of developing of urologic cancer [HRs (95% CI) = 1.35 (1.10–1.66)] and kidney cancer [HR (95% CI) = 1.74 (1.21–2.51)], while central obesity increased the risk of developing prostate cancer [HR (95% CI) = 1.68 (1.18–2.40)].

**Conclusions:**

MetS increased the risk of developing urologic, kidney, and prostate cancers but had no association with the development of bladder cancer.

## Introduction

Worldwide, urologic malignancies pose a serious threat to human health. Based on GLOBOCAN data, in 2020 alone, the number of new cases of prostate, bladder, and kidney cancers reached 1,414,259, 573,278, and 431,288, respectively. Meanwhile, in the same year, 375,304, 212,536, and 179,368 deaths resulted from prostate, bladder, and kidney cancers, respectively [1]. Although urologic cancers are highly dangerous, their etiology remains unclear, with known risk factors including age, race, family history of malignancy, and smoking [2–4].

Metabolic syndrome (MetS) is a comprehensive syndrome involving blood pressure, glucose and lipids abnormalities, and central obesity. MetS is associated with an increased risk of colorectal, endometrial, and postmenopausal breast cancer in humans [5]; however, its association with urologic cancers remains controversial. Some studies have found that MetS is associated with an increased risk of bladder, prostate, and kidney cancers [6–9], while other studies have produced contradictory results [10, 11]. Previous studies have only investigated the association between MetS and the risk of single-site urologic cancers; its association with overall urologic cancers has not yet been reported. Moreover, these past studies include non-cohort studies [6, 8, 9] and cohort studies with short median or mean follow-up times (5, 6.9, and 2.7 years) [7, 10, 11], preventing them from accurately assessing the association between MetS and the risk of developing urologic cancers. To clarify whether MetS is associated with urologic cancers, we analyzed the effect of MetS on urologic cancers using the population from the Kailuan Study.

## Population and methods

### Study cohort

The Kailuan Study is an ongoing observational cohort study of a functional community population that began in 2006. The MetS information of the Kailuan Study subjects was obtained at baseline (from May 2006 to December 2007), and over 10 years of follow-up data were obtained for events including urologic cancers, allowing us to study the association between MetS and urologic cancers. More details about the Kailuan Study can be found in the literature [12, 13]. The inclusion criteria for this study were as follows: (1) included in the baseline survey population (age ≥ 18 years) of the Kailuan Study; (2) complete data on waist circumference (WC), blood pressure (BP), fasting blood glucose (FBG), triglycerides (TG), and high-density lipoprotein cholesterol (HDL-C); and (3) signed the informed consent form. The exclusion criterion was a history of malignancy. The study was conducted in accordance with the Declaration of Helsinki and was approved by the Ethics Committee of Kailuan General Hospital.

### Collection of exposure information

The baseline data included sociodemographic characteristics (age, gender, occupation, education, economic income, and marital status), lifestyle characteristics (smoking, alcohol consumption, salt intake, and physical activity), history of previous diseases, physical examination data (WC and BP), and blood indices (FBG and lipids). WC was measured at the level of the midpoint between the anterior superior iliac crest and the lower rib cage. Systolic and diastolic blood pressures were measured using a mercury sphygmomanometer with a suitable cuff on the left arm of the subject after 5 min of rest and then again after 5 min; the average of the two measurements was recorded. Early-morning fasting blood samples were collected from the subjects to measure blood glucose and lipids. The FBG level was measured using the hexokinase/glucose-6-phosphate dehydrogenase method, and the coefficient of variance of blind quality control samples was < 2.0%. TG were determined by glycerol phosphate oxidase assay (coefficient of mutual variation < 10%). After the precipitation of apolipoprotein B with dextrose sulfate and magnesium chloride, the HDL-C level was measured in the supernatant [13]. The above measurements were conducted by well-trained physicians or nurses using a standardized protocol.

### Definition of variables

According to the harmonized International Diabetes Federation criteria [14], MetS is defined as the occurrence of three or more of the following five risk factors: (1) central obesity, defined as WC ≥ 90 cm in men and WC ≥ 80 cm in women; (2) elevated TG, defined as TG ≥ 150 mg/dL (1.7 mmol/L) and/or drug treatment for elevated TG; (3) reduced HDL-C, defined as HDL-C < 40 mg/dL (1.0 mmol/L) in males and HDL-C < 50 mg/dL (1.3 mmol/L) in females and/or drug treatment for reduced HDL-C; (4) elevated BP, defined as systolic BP ≥ 130 mmHg and/or diastolic BP ≥ 85 mmHg, and/or a history of hypertension treated with antihypertensive drugs; and (5) elevated FBG, defined as FBG ≥ 100 mg/dL (5.6 mmol/L) and/or receiving glucose-lowering medication for elevated glucose.

Smoking status was classified as follows: non-smoker (has never smoked); former smoker (has not smoked for more than 12 months); and current smoker (smokes one or more cigarettes per week for not less than 12 consecutive months). Alcohol consumption status was divided into the following categories: never, former (abstained from alcohol for more than six months) and current alcohol consumption (one or more drinks per month for no less than six months in a row). Physical activity was classified according to the frequency of physical activity (20 min = 1 instance of activity) performed during leisure time. The categories of physical activity were: no exercise, exercise occasionally (between one and three instances of activity per week), and frequent exercise (four or more instances of activity per week).

### Collection of endpoint event information

The follow-up period started when the observation subjects completed the baseline examination. The last follow-up was conducted on December 31, 2019. The follow-up endpoint event was a new urologic cancer or death in the observed subject (whichever came first). First, information on the observation subjects’ medical visits was obtained through the Tangshan City health insurance system. Professionally trained investigators then went to the hospitals to collect information on the subjects’ medical history. Clinicians verified the pathology, imaging (including magnetic resonance imaging, computed tomography, and color Doppler ultrasonography), and blood biochemical examination results to confirm and refine the diagnosis of urologic cancers. Tumor cases were classified according to the International Classification of Diseases-10 (ICD-10). Urologic cancers include prostate cancer, kidney cancer, carcinoma renal pelvis, ureteral cancer, bladder cancer, and urethral cancer with codes C61 and C64–C68, respectively. Information on fatal events was obtained through the Kailuan Group social insurance system.

### Statistical methods

Statistical analysis of the data was performed using SAS 9.4. Continuous variables were expressed as mean ± standard deviation, while categorical variables were expressed as the number of cases (percentage). Differences in baseline demographic characteristics between the MetS and non-MetS groups were analyzed using independent-sample t-test and chi-square test, respectively. The Kaplan–Meier method was used to calculate the cumulative incidence of urologic cancers and kidney, prostate, and bladder cancers in the MetS and non-MetS groups. The difference in cumulative incidence between the two groups was compared by log-rank test. Multifactorial Cox proportional hazards modelling was applied to analyze the associations between MetS, the number of MetS components, and single components of MetS and the risk of developing urologic cancers as well as kidney, prostate, and bladder cancers. We stratified the study populations by age (four sections), gender, and occupation to test robustness of results within subgroups. Interactions effect measure on multiplicative scale were evaluated by additionally including the product term of age, gender, or occupation with MetS in models. Sensitivity analysis was performed by applying Cox proportional hazards modellings after excluding participants with a history of myocardial infarction and stroke as well as cases with new urologic cancers within two years of the start of the follow-up period. All statistical tests were considered statistically significant at *P* < 0.05 (two-sided).

## Results

### Baseline characteristics

The number of participants in the 2006 baseline survey was 101,510, of which 98,348 met the inclusion criteria for this study. A total of 373 individuals with a history of malignancy were excluded, and 97,975 participants (78,345 males and 19,630 females) were eventually included in the statistical analysis. The mean age of the observed subjects was 51.76 ± 12.56 years. The MetS and non-MetS groups included 31,359 and 66,616 subjects, respectively. Compared to the non-MetS group, the mean age and the proportions of subjects with heavy salt intake, males, white-collar workers, low education level, and low income were higher in the MetS group, whereas the proportions of non-smokers and non-drinkers were lower (all *P* values < 0.05; Table 1).

**Table 1:** Baseline characteristics of the participants by MetS status

Characteristics	Total cohort (*n* = 97,975)	Non-MetS (*n* = 66,616)	MetS (*n* = 31,359)	*p*
Age(years,mean ± SD)	51.76 ± 12.56	50.46 ± 12.86	54.52 ± 11.41	< 0.001
Gender, n(%)				
Female	19,630(20.04)	13,982(20.99)	5648(18.01)	< 0.001
Male	78,345(79.96)	52,634(79.01)	25,711(81.99)	
Smoking status, n(%)				
Never	58,457(59.67)	40,268(60.45)	18,189(58.00)	< 0.001
Former	5772(5.89)	3398(5.10)	2374(7.57)	
Current	33,746(34.44)	22,950(34.45)	10,796(34.43)	
Alcohol consumption, n(%)				
Never	57,542(58.73)	39,384(59.12)	18,158(57.90)	< 0.001
Former	3906(3.99)	2309(3.47)	1597(5.09)	
Current	36,527(37.28)	24,923(37.41)	11,604(37.01)	
Occupation, n(%)				
White collar	7919(8.08)	5284(7.93)	2635(8.40)	0.012
Blue collar	90,056(91.92)	61,332(92.07)	28,724(91.60)	
Education level, n(%)				
Illiteracy and primary	11,134(11.36)	6782(10.18)	4352(13.88)	< 0.001
Middle school	79,964(81.62)	54,599(81.96)	25,365(80.89)	
College and above	6877(7.02)	5235(7.86)	1642(5.23)	
Income(yuan per psrson per month), n(%)				
< 600	28,419(29.01)	18,886(28.35)	9533(30.40)	< 0.001
≥ 600- < 1000	62,982(64.28)	43,237(64.90)	19,745(62.96)	
≥ 1000	6574(6.71)	4493(6.75)	2081(6.64)	
Marital status, n(%)				
Single	4503(4.60)	3238(4.86)	1265(4.03)	< 0.001
Married/cohabiting	93,472(95.40)	63,378(95.14)	30,094(95.97)	
Salt intake, n(%)				
Light	9149(9.34)	6203(9.31)	2946(9.39)	< 0.001
General	78,257(79.87)	53,714(80.63)	24,543(78.26)	
Heavy	10,569(10.79)	6699(10.06)	3870(12.35)	
Physical activities, n(%)				
Never	8666(8.85)	5901(8.86)	2765(8.82)	< 0.001
Occasionally	73,797(75.32)	51,011(76.57)	22,786(72.66)	
Frequently	15,512(15.83)	9704(14.57)	5808(18.52)	

*MetS* metabolic syndrome

### Incidence of urologic cancers in the MetS and non-MetS groups

**Fig. 1:**
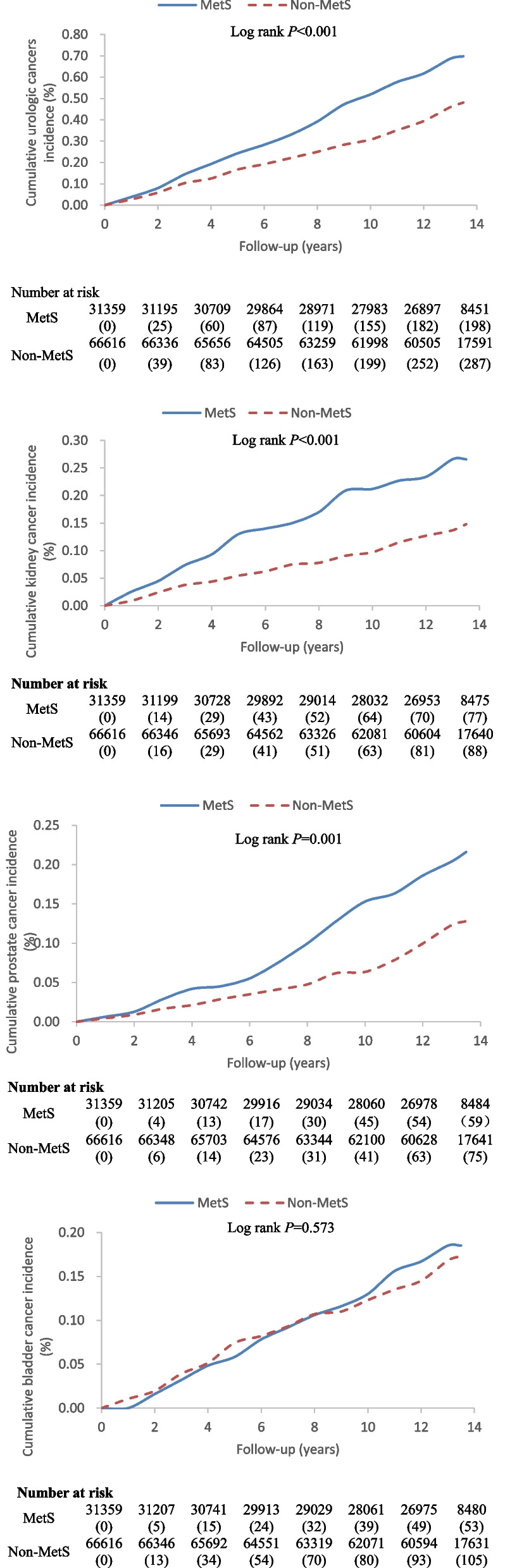
Cumulative incidence of urologic, kidney, prostate, and bladder cancers by MetS status

The cumulative follow-up period in this study was 1,209,178.65 person-years, with a median follow-up time of 13.03 years. A cumulative total of 485 new urologic cancers (*n* = 198 in the MetS group; *n* = 287 in the non-MetS group) were detected during the follow-up period, including 165 cases of kidney cancer (*n* = 77 in the MetS group; *n* = 88 in the non-MetS group), 134 cases of prostate cancer (MetS group *n* = 59; non- MetS group *n* = 75), 158 cases of bladder cancer (*n* = 53 in the MetS group; *n* = 105 in the non-MetS group), and 28 cases of other urologic cancers (*n* = 9 in the MetS group; *n* = 19 in the non-MetS group). The cumulative incidence rates in the MetS and non-MetS groups were 0.70% and 0.48% for all urologic cancers, 0.27% and 0.15% for kidney cancer, 0.22% and 0.13% for prostate cancer, and 0.19% and 0.17% for bladder cancer, respectively. The differences in the cumulative incidences of urologic cancers, kidney cancer, and prostate cancer were statistically different between the two groups based on by log-rank test, whereas the difference in the cumulative incidence of bladder cancer was not statistically significant (Fig. 1).

### Association between MetS and risk of urologic cancers

Cox proportional hazards modelling was performed with the occurrence of urologic, kidney, and prostate cancers as the dependent variables and the presence of MetS as the independent variable. Model 1 was adjusted for age and gender, while model 2 was adjusted for smoking, alcohol consumption, occupation, education, income level, marital status, salt intake, and physical activity based on model 1. The hazard ratios (HRs) and 95% confidence intervals (95% CIs) for urologic cancer, kidney cancer, and prostate cancer in the MetS group compared with the non-MetS group were 1.29 (1.08–1.55), 1.74 (1.28–2.37), and 1.47 (1.04– 2.07), respectively. When different numbers of MetS components were used as independent variables, Cox proportional hazards modelling showed that the risk of kidney cancer was significantly increased in subjects with three and four/five MetS components compared with subjects without any MetS components [HRs (95% CIs) = 1.96 (1.03–3.75) and 2.82 (1.46–5.47), respectively] (Table 2).

**Table 2:** Associations between MetS and urologic, kidney, prostate, and bladder cancer risk

Groups		Total cases	Person years	Incident cases	Model 1	Model 2
					**HR(95%CI)**	**HR(95%CI)**
**Urologic cancer**						
MetS status	Non-MetS	66,616	828,271.46	287	Ref	Ref
	MetS	31,359	380,907.19	198	1.28(1.07–1.54)	1.29(1.08–1.55)
No. of MetS components	0	12,979	164,307.06	43	Ref	Ref
	1	25,627	318,630.72	105	0.98(0.69–1.40)	0.99(0.69–1.41)
	2	28,010	345,333.67	139	1.07(0.76–1.50)	1.08(0.77–1.53)
	3	19,938	243,003.49	122	1.28(0.90–1.81)	1.30(0.92–1.84)
	4–5	11,421	137,903.70	76	1.38(0.95–2.00)	1.39(0.96–2.03)
	*p*_trend_				0.008	0.007
**Kidney cancer**						
MetS status	Non-MetS	66,616	829,139.77	88	Ref	Ref
	MetS	31,359	381,403.48	77	1.74(1.28–2.36)	1.74(1.28–2.37)
No. of MetS components	0	12,979	164,421.30	12	Ref	Ref
	1	25,627	318,972.08	30	1.15(0.59–2.24)	1.16(0.59–2.27)
	2	28,010	345,746.39	46	1.53(0.81–2.90)	1.56(0.82–2.95)
	3	19,938	243,335.17	42	1.93(1.01–3.69)	1.96(1.03–3.75)
	4–5	11,421	138,068.32	35	2.79(1.44–5.41)	2.82(1.46–5.47)
	*p*_trend_				< 0.001	< 0.001
**Prostate cancer**						
MetS status	Non-MetS	66,616	829,318.96	75	Ref	Ref
	MetS	31,359	381,647.01	59	1.47(1.04–2.06)	1.47(1.04–2.07)
No. of MetS components	0	12,979	164,450.99	11	Ref	Ref
	1	25,627	319,007.40	28	0.92(0.46–1.86)	0.92(0.46–1.84)
	2	28,010	345,860.57	36	0.96(0.49–1.89)	0.95(0.48–1.86)
	3	19,938	243,468.49	39	1.44(0.74–2.82)	1.43(0.73–2.80)
	4–5	11,421	138,178.52	20	1.31(0.63–2.73)	1.30(0.62–2.71)
	*p*_trend_				0.085	0.088
**Bladder cancer**						
MetS status	Non-MetS	66,616	829,035.72	105	Ref	Ref
	MetS	31,359	381,627.63	53	0.93(0.67–1.29)	0.93(0.67–1.30)
No. of MetS components	0	12,979	164,396.49	17	Ref	Ref
	1	25,627	318,911.15	39	0.87(0.49–1.55)	0.89(0.50–1.57)
	2	28,010	345,728.08	49	0.89(0.51–1.55)	0.91(0.52–1.58)
	3	19,938	243,447.21	37	0.92(0.51–1.63)	0.94(0.53–1.67)
	4–5	11,421	138,180.42	16	0.69(0.35–1.37)	0.70(0.35–1.39)
	*p*_trend_				0.444	0.479

Model 1 was adjusted for age and gender; model 2 was further adjusted for smoking status, alcohol consumption, occupation, education level, income, marital status, salt intake, and physical activity

*MetS* metabolic syndrome, *No.*, number

### Association between MetS components and risk of urologic cancers

By setting subjects with normal BP, FBG, HDL-C, TG, and WC as the controls, Cox proportional hazards modelling showed that elevated BP increased the risk of urologic and kidney cancers [HRs (95% CI) = 1.35 (1.10–1.66) and 1.74 (1.21–2.51), respectively]. Meanwhile, central obesity increased the risk of prostate cancer [HR (95%CI) = 1.68 (1.18–2.40)] (Table 3).

**Table 3:** Associations between individual components of MetS and urologic, kidney, prostate, and bladder cancer risk

Variable	Urologic cancer	Kidney cancer	Prostate cancer	Bladder cancer
	**HR(95%CI)**	**HR(95%CI)**	**HR(95%CI)**	**HR(95%CI)**
BP(mmHg)^a^				
Normal	Ref	Ref	Ref	Ref
High	1.35(1.10–1.66)	1.74(1.21–2.51)	1.24(0.83–1.87)	1.13(0.79–1.61)
FBG(mmol/l)^b^				
Normal	Ref	Ref	Ref	Ref
High	0.96(0.79–1.16)	1.09(0.79–1.51)	0.89(0.61–1.29)	1.00(0.71–1.40)
HDL-C(mmol/l)^c^				
Normal	Ref	Ref	Ref	Ref
Low	0.91(0.74–1.12)	1.37(0.99–1.90)	0.78(0.52–1.17)	0.58(0.39–0.88)
TG(mmol/l)^d^				
Normal	Ref	Ref	Ref	Ref
High	1.11(0.92–1.35)	1.22(0.88–1.68)	1.09(0.75–1.58)	1.12(0.79–1.58)
WC(cm)^e^				
Normal	Ref	Ref	Ref	Ref
High	1.20(1.00–1.44)	1.19(0.86–1.64)	1.68(1.18–2.40)	0.93(0.67–1.29)

Adjusted for all factors (age, gender, smoking status, alcohol consumption, occupation, education level, income, marital status, salt intake, physical activities, BP, FBG, HDL-C, TG, and WC) excluded itself

*MetS* metabolic syndrome, *BP* blood pressure, *FBG* fasting blood glucose, *HDL-C* high-density lipoprotein cholesterol, *TG* triglycerides, *WC* waist circumference

^a^High was defined as systolic blood pressure ≥ 130 mmHg and/or diastolic blood pressure ≥ 85 mmHg, and/or a history of hypertension treated with antihypertensive drugs. Normal was defined as systolic blood pressure < 130 mmHg and diastolic blood pressure < 85 mmHg

^b^High was defined as FBG ≥ 100 mg/dL (5.6 mmol/L) and/or receiving glucose-lowering medication for elevated glucose. Normal was defined as FBG < 100 mg/dL (5.6 mmol/L)

^c^Low was defined as HDL-C < 40 mg/dL (1.0 mmol/L) in males and HDL-C < 50 mg/dL (1.3 mmol/L) in females and/or drug treatment for reduced HDL-C.Normal was defined as HDL-C ≥ 40 mg/dL (1.0 mmol/L) in males and HDL-C ≥ 50 mg/dL (1.3 mmol/L) in females and/or drug treatment for reduced HDL-C

^d^High was defined as TG ≥ 150 mg/dL (1.7 mmol/L) and/or drug treatment for elevated TG. Normal was defined as TG < 150 mg/dL (1.7 mmol/L)

^e^High was defined as WC ≥ 90 cm in males and WC ≥ 80 cm in females. Normal was defined as WC < 90 cm in males and WC < 80 cm in females

### Subgroup analysis of MetS and the risk of urologic cancers

Cox proportional hazards modellings were used to analyze the interactions among covariates (age, gender, and occupation) and MetS. In the male subgroup and 45–54 year age subgroup, the analysis revealed an increased risk of urologic and kidney cancers in patients with MetS. The analysis also indicated an increased risk of kidney cancer in subjects with MetS and under 45 years old. The risk of urologic, kidney, and prostate cancers was elevated among blue-collar workers with MetS (Table 4).

**Table 4:** Subgroup analysis of the associations between MetS and urologic, kidney, prostate, and bladder cancer risk

Variable	Urologic cancer		Kidney cancer		Prostate cancer		Bladder cancer	
	**HR (95%CI)**	***P* for inter-action**	**HR (95%CI)**	***P* for Inter-action**	**HR (95%CI)**	***P* for inter-action**	**HR (95%CI)**	***P* for inter-action**
Age(years)		0.102		0.182		0.678		0.511
< 45	1.98 (0.96–4.08)		2.54 (1.07–6.06)		-		1.63 (0.39–6.91)	
45–54	1.48 (1.02–2.16)		1.82 (1.13–2.95)		1.61 (0.54–4.79)		0.87 (0.38–2.01)	
55–64	1.13 (0.83–1.55)		1.34 (0.77–2.32)		1.39 (0.81–2.41)		0.97 (0.54–1.73)	
≥ 65	1.15 (0.85–1.56)		1.47 (0.67–3.23)		1.35 (0.84–2.18)		0.81 (0.50–1.32)	
*P*_trend_	< 0.001		0.033		< 0.001		< 0.001	
Gender		0.165		0.040				0.097
Female	0.77 (0.34–1.77)		0.49 (0.16–1.56)		-		3.44 (0.60–19.65)	
Male	1.33 (1.10–1.60)		1.96 (1.42–2.71)		-		0.87 (0.62–1.22)	
Occupation		0.004		0.049		0.363		0.228
White collar	0.41 (0.19–0.91)		0.28 (0.06–1.29)		0.87 (0.25–2.99)		0.33 (0.07–1.58)	
Blue collar	1.40 (1.16–1.69)		1.98 (1.44–2.72)		1.54 (1.08–2.20)		0.98 (0.70–1.38)	

Compared with the non-MetS group respectively

*MetS* metabolic syndrome

Adjusted for age, gender, smoking status, alcohol consumption, occupation, education level, income, marital status, salt intake, and physical activity

The interaction terms were age, gender, and occupation with MetS, respectively

### Sensitivity analysis of the relationship between MetS and the risk of developing urologic cancers

In the sensitivity analysis, excluding the subjects with a history of myocardial infarction and stroke, the overall risk of developing urologic cancers and the specific risks for developing kidney and prostate cancers were higher in the MetS group compared with the non-MetS group, with HRs (95% CIs) of 1.32 (1.10–1.60), 1.82 (1.32–2.51), and 1.50 (1.06–2.13), respectively. The HRs (95% CIs) for kidney cancer in subjects with three and four/five MetS components compared to those without any MetS components were 2.18 (1.12–4.26) and 2.91 (1.45–5.82), respectively. When excluding participants who developed urologic cancers within two years after the start of the follow-up period, the risk of urologic cancers as well as the risks of kidney and prostate cancers were significantly higher in the MetS group, with HRs (95% CIs) of 1.31 (1.08–1.59), 1.77 (1.26–2.48), and 1.49 (1.05–2.13), respectively. The HR (95% CI) for kidney cancer in subjects with four/five MetS components compared to subjects without any MetS components was 3.01 (1.51–6.00) (Table 5).

**Table 5:** Sensitivity analyses of the associations between MetS and urologic, kidney, prostate, and bladder cancer risk

Groups		Urologic cancer	Kidney cancer	Prostate cancer	Bladder cancer
		**HR(95%CI)**	**HR(95%CI)**	**HR(95%CI)**	**HR(95%CI)**
**Excluding participants with history of myocardial infarction and stroke**					
MetS status	Non-MetS	Ref	Ref	Ref	Ref
	MetS	1.32(1.10–1.60)	1.82(1.32–2.51)	1.50(1.06–2.13)	0.92(0.65–1.31)
No. of MetS components	0	Ref	Ref	Ref	Ref
	1	0.98(0.68–1.40)	1.11(0.55–2.26)	0.98(0.48–2.03)	0.84(0.47–1.49)
	2	1.10(0.77–1.57)	1.68(0.86–3.26)	1.07(0.53–2.16)	0.85(0.48–1.49)
	3	1.34(0.93–1.91)	2.18(1.12–4.26)	1.60(0.79–3.21)	0.85(0.47–1.54)
	4–5	1.44(0.97–2.11)	2.91(1.45–5.82)	1.45(0.67–3.13)	0.70(0.35–1.41)
	_*P*trend_	0.003	< 0.001	0.050	0.446
**Excluding participants who developed urologic cancer within the first 2 years of follow-up**					
MetS status	Non-MetS	Ref	Ref	Ref	Ref
	MetS	1.31(1.08–1.59)	1.77(1.26–2.48)	1.49(1.05–2.13)	0.97(0.68–1.37)
No. of MetS components	0	Ref	Ref	Ref	Ref
	1	1.00(0.69–1.46)	1.08(0.53–2.19)	0.98(0.47–2.02)	0.96(0.52–1.75)
	2	0.97(0.68–1.40)	1.37(0.69–2.70)	0.93(0.46–1.90)	0.84(0.46–1.53)
	3	1.21(0.83–1.75)	1.59(0.79–3.18)	1.46(0.72–2.96)	0.95(0.51–1.76)
	4–5	1.45(0.98–2.15)	3.01(1.51–6.00)	1.36(0.63–2.94)	0.75(0.37–1.54)
	_*P*trend_	0.012	< 0.001	0.095	0.501

Adjusted for age, gender, smoking status, alcohol consumption, occupation, education level, income, marital status, salt intake, and physical activity

*MetS* metabolic syndrome, *No.* number

## Discussion

The results of this study indicate that MetS increases the risk of developing urologic cancers as well as kidney and prostate cancers, and the risk of developing urologic cancers and kidney cancers increases as the number of MetS components in the subject increases. Elevated BP and central obesity were identified as independent risk factors for urologic cancer, kidney cancer, and prostate cancer.

We found that MetS increased the risk of developing urologic cancer by 29% compared with the non-MetS population. Compared with individuals without MetS components, the risk of urologic cancer increased as the number of MetS components present in the individual increased (*P*_trend_ = 0.007), especially in patients with kidney cancer (*P*_trend_ < 0.001). This suggests that MetS is not only a risk factor for the development of urologic cancers, but that this risk is correlated with the number of MetS components.

Although MetS increases the risk of developing urologic cancers, the different MetS components vary in terms of their contributions to increasing the risk of developing urologic cancers. High BP is a factor in the increased risk of developing overall urologic cancers along with kidney cancer. Although no previous studies have linked high BP to the overall risk of urologic cancers, a Korean cohort study [15] found that the risk of developing kidney cancer was higher in hypertensive subjects than in non-hypertensive subjects, and that the risk of developing kidney cancer increased significantly with elevated systolic or diastolic BP in a dose-dependent manner. The present study indicated a significantly higher risk of prostate cancer in centrally obese individuals [HR (95% CI) = 1.68 (1.18–2.40)], consistent with the findings of Boehm et al. [16], who reported that abdominal obesity is a predictor of developing prostate cancer. However, other studies have found no association [17] or even a negative correlation [18] between the two.

We found that the increased risk of developing urologic cancers due to MetS was site specific. MetS significantly elevated the risk of developing kidney cancer, and the risk increased with the number of abnormal MetS components, consistent with the findings of Me-Can [19] and Turkey [8]. However, in an Italian study, Russo et al. found that MetS was not associated with the risk of kidney cancer development [11]. Likewise, we found that MetS increased the risk of prostate cancer development, consistent with previous findings [7, 20–22]. While several study showed that metabolic syndrome was associated with high-grade prostate cancer [23–25], but not overall prostate cancer or low grade prostate cancer [23]. And there were significant differences in treatment patterns and health-related quality of life after treatment of different race patients with prostate cancer [26]. Metabolic health factors in patients with prostate cancer can be improved by periodic fasting mimicking diet [27]. However, we did not find an association between MetS and the risk of developing bladder cancer. These differences in findings may be related to differences in the races of the subjects, the length of the follow-up period, and the MetS diagnostic criteria.

Subgroup analysis showed that the effect of MetS on urologic, kidney, prostate, and bladder cancers was negatively correlated with age (*P*_trend_ < 0.05). MetS increased the risk of developing urologic cancers in subjects aged 45–54 years, while the effect of MetS on kidney cancer was more significant in younger subgroups (< 45 and 45–54 years old). The analysis of gender and occupational subgroups showed an increased risk of developing urologic cancer as well as kidney cancer in the male population with MetS and an increased risk of developing urologic cancer as well as kidney and prostate cancers in blue collars with MetS. These findings suggest that the prevention and control of MetS should be prioritized in the abovementioned groups to reduce the occurrence of urologic cancers.

In the sensitivity analysis, after excluding subjects with a history of myocardial infarction and stroke, the risk of developing urologic cancers as well as kidney and prostate cancers was significantly increased in the MetS group. This may be related to the control of MetS components through lifestyle and dietary changes in this group; particularly, this population tends to take statins and aspirin for long periods of time, and statins have been reported to reduce the risk for developing kidney [28] and prostate [29] cancers. Long-term aspirin use to prevent cardiovascular disease may also reduce the risk of developing cancer overall [30] and prostate cancer specifically [31].

Although the association between MetS and cancer risk is not fully understood [32–34], they share many common risk factors, including older age, obesity, lack of exercise, unhealthy diet, disturbance of the biological clock, oxidative stress, air pollution, and damage caused by exposure to substances that disrupt the endocrine system [35–38]. Several pathophysiological mechanisms may tentatively explain the relationship between MetS and cancer development. First, MetS is often characterized by insulin resistance and associated hyperinsulinemia[39]. Insulin resistance stimulates the production of reactive oxygen species, which can damage DNA and promote malignant transformation [40]. Hyperinsulinemia increases the biological activity of IGF-1 [41], which induces and activates the Ras/Raf/MAPK and PI3K/Akt/mTOR pathways, thus reduces apoptosis promotes cell proliferation and survival, and increases the risk of tumor development [42]. Second, chronic hyperglycemia can also cause oxidative damage to cellular DNA [43], leading to worse tumor grading, greater metastatic potential, and chemotherapy resistance [44]. Obesity can lead to the infiltration of immune cells such as macrophages and lymphocytes, which are important sources of circulating pro-inflammatory factors (tumor necrosis factor-α and interleukin-6); high levels of pro-inflammatory mediators promote cancer development and progression through local and systemic effects [33, 45–47]. The obese state is characterized by a high leptin-adiponcetin ratio, with adiponcetin having an inhibitory effect on cell proliferation and metastasis, and leptin stimulating cell proliferation and promoting invasion and migration [48]. In addition, elevated angiogenic factors in hypertensive patients may be associated with the risk of developing renal malignancies [49].

The strengths of this study are the large sample size, the prospective cohort design, and the robust follow-up mechanism. This study also has some limitations. We used only baseline MetS data to analyze the association between MetS and urologic cancer risk; time-dependent exposures during follow-up were not considered. Changes in MetS status or the MetS composition over time may affect the risk of developing urologic cancers and thus should be explored in depth in subsequent studies.

In conclusion, we found that the presence of MetS is associated with the risk of kidney and prostate cancers. In addition, elevated BP and central obesity were independent risk factors for kidney cancer and prostate cancer, respectively. In clinical practice, we expect that the risk of kidney and prostate cancers could be reduced by correcting the MetS status and reducing the number of MetS components, particularly maintaining BP and WC within the appropriate ranges.

## Data Availability

All data generated or analysed during this study are included in this published article.
